# Effects of Four Antibiotics on the Diversity of the Intestinal Microbiota

**DOI:** 10.1128/spectrum.01904-21

**Published:** 2022-03-21

**Authors:** Ce Huang, Shengyu Feng, Fengjiao Huo, Hailiang Liu

**Affiliations:** a Institute for Regenerative Medicine, Shanghai East Hospital, Tongji University School of Medicine, Shanghai, China; b Key Laboratory of Xinjiang Phytomedicine Resource and Utilization, Ministry of Education, College of Life Sciences, Shihezi University, Shihezi, China; The Pennsylvania State University

**Keywords:** intestinal microbiota, ampicillin, vancomycin, metronidazole, neomycin

## Abstract

Oral antibiotics remain the therapy of choice for severe bacterial infections; however, antibiotic use disrupts the intestinal microbiota, increasing the risk of colonization by intestinal pathogens. Currently, our understanding of antibiotic-mediated disturbances of the microbiota remains at the level of bacterial families or specific species, and little is known about the effect of antibiotics on potentially beneficial and pathogenic bacteria under the conditions of gut microbiota dysbiosis. Additionally, the question of whether the effects of antibiotics on the gut microbiota are temporary or permanent is controversial. In this study, we used 16S rRNA gene sequencing to evaluate the short- and long-term effects of ampicillin, vancomycin, metronidazole, and neomycin on the murine intestinal microbiota. We found that the changes in the intestinal microbiota reflected the antibiotics’ mechanisms of action and that dysbiosis of the intestinal microbiota led to competition between different bacterial communities. In particular, an increase in *Enterococcus*, which accompanies a decrease in probiotics-related genera such as *Lactobacillus*, is commonly seen across antibiotic treatments. In addition, we found that these oral antibiotics had long-term negative effects on the intestinal microbiota and promoted the development of antibiotic-resistant bacterial strains. These results indicate that ampicillin, vancomycin, metronidazole, and neomycin have long-term negative effects and can cause irreversible changes in the diversity of the intestinal microbiota, thereby increasing the risk of host disease.

**IMPORTANCE** The intestinal microbiota is a dynamic community of hundreds of millions of microorganisms that play important roles in human health. However, treatment with antibiotics can disrupt the delicate balance of this community, leading to deleterious effects on the host such as inflammation and enhanced susceptibility to infection. To date, most studies of the effects of antibiotic treatment on the microbiota have focused on specific intestinal pathogens and bacterial families. However, few studies have investigated the effects of antibiotic treatment on the relative abundance of probiotic bacteria, pathogenic bacteria, and opportunistic bacterial pathogens in the gut.

## INTRODUCTION

The host intestinal tract provides the intestinal microbiota with an anaerobic, or hypoxic, nutrient-rich environment and, as a result, hundreds of millions of different types of microorganisms parasitize the human intestine, forming a complex ecological environment (1). Microorganisms within the intestinal microbiota can be divided into three categories based on their interaction with the host: mutualists (benefiting themselves and the host), commensals (benefiting themselves but not the host), and pathogens (benefiting themselves by harming the host) (2). Here, we will refer to mutualists as “potentially beneficial bacteria.” Potentially beneficial bacteria play a very important role in maintaining the stability of the intestinal microbiota, and they also provide the host with biochemical metabolic pathways and enzymes which it does not already have, help the host to break down nutrients, synthesize nutrients needed by the human body, maintain the stability of the nervous system, activate the immune system, and maintain intestinal homeostasis (3, 4). For example, *Turicibacter*, *Coprobacillus*, and *Lactobacillus* produce lactic acid through glycolysis, which can provide intestinal epithelial cells with nutrition and regulate immune function (5–7), and *Parabacteroides*, *Faecalibacterium*, *Odoribacter*, and *Coprococcus* can reduce intestinal inflammation by secreting acetic acid and butyrate, thereby decreasing the incidence of inflammatory colitis, Crohn’s disease, and other illnesses (8–11). *Roseburia* and *Akkermansia* can ferment a variety of carbohydrates and have been used to treat diseases such as obesity and diabetes (12, 13). Pathogenic bacteria account for a small proportion of the microorganisms in the intestine. Many of these species, when present in small quantities, are an important part of a healthy intestinal microbiota. However, when intestinal microbiota dysbiosis occurs, these pathogens will harm the host. Among them, *Enterococcus*, Enterobacter, and Clostridium perfringens are the main pathogenic bacteria. They break down some components of food into amines through their own unique biochemical metabolic pathways. However, they also produce indole, phenols, and unique microbial toxins, which cause certain intestinal diseases (14, 15). For example, Clostridium difficile growth and reproduction can cause diarrhea and pseudomeningitis (16), and an excessive number of enterococci can cause abdominal and pelvic infections (17). When the number of potentially beneficial bacteria in the human intestine decreases and the number of pathogenic bacteria increases, intestinal homeostasis is disrupted, which can cause host metabolic disorders and affect the host’s immune system.

Antibiotics are frequently used to treat bacterial infections. Epidemiological studies have shown that 71% of intensive care unit patients are on antibiotics. Long-term, excessive use of antibiotics can cause serious adverse consequences. Although antibiotics kill bacteria and inhibit their growth, they can also induce drug resistance. In addition, antibiotic use can temporarily or permanently alter the composition of the intestinal microbiota, promote colonization by intestinal pathogens, and trigger the development of some intestinal diseases (18, 19). In recent years, disruption of the intestinal microbiota by antibiotics has received increasing attention. Some studies have demonstrated a direct link between antibiotic use and alterations in the intestinal microbiota. For example, short-term treatment of mice with metronidazole, vancomycin, and clindamycin can reduce their susceptibility to infection with C. difficile, vancomycin-resistant *Enterococci*, Klebsiella pneumonia, and Escherichia coli (20, 21). In this study, we investigated the effects of four commonly used antibiotics: vancomycin, which is typically used to treat infections with Gram-positive bacteria; ampicillin and neomycin, which are typically used for Gram-negative bacterial infections; and metronidazole, which is typically used to treat anaerobic bacterial infections, to account for most of the bacterial components of the intestinal microbiota. To date, studies of the relationship between these antibiotics and the intestinal microbiota have mostly focused on the impact of antibiotics on colonization by potentially pathogenic bacteria such as C. difficile and Enterobacter (21). In addition, these studies of microbiota disruption associated with antibiotic use have remained at the level of bacterial families or specific species (22–24). Combined treatment with ampicillin, vancomycin, neomycin, and metronidazole provides bactericidal activity against the full spectrum of bacteria and, notably, dual activity against both Gram-positive (ampicillin and vancomycin) and Gram-negative (ampicillin and neomycin) aerobic and facultative strains (see Table S3 in the supplemental material). Few studies have used high-throughput 16s rRNA gene sequencing technology to conduct in-depth analysis of changes in intestinal microbiota diversity in response to treatment with these antibiotics, and especially to quantitatively assess the relative proportions of potentially beneficial bacteria and potentially pathogenic bacteria.

On the other hand, a number of recent studies have reported the use of a combination of four antibiotics added to drinking water to eliminate the gut microbiota, reducing the fecal DNA content to 3% that of the control (25–27). However, most of these studies only quantified the total DNA content of the feces, which demonstrated that the gut microbiota was reduced, but did not provide any detailed information regarding the effect of this treatment regimen on intestinal microbiota dysbiosis. There is also a lack of research on whether this combination of antibiotics can minimize the diversity of intestinal microbiota compared with single antibiotic use.

In this study, we used 16S rRNA gene sequencing technology to analyze the short- and long-term effects of ampicillin, vancomycin, metronidazole, and neomycin on the murine intestinal microbiota. Additionally, we estimated the relative numbers of bacteria with beneficial and pathogenic potential by comparing relative numbers between genera containing multiple probiotic bacteria, such as *Lactobacillus* and *Bifidobacterium*, and those containing multiple pathogenic bacteria, such as *Enterococcus* and *Bacillus*, at the genus level. In addition, we compared a mixture of four antibiotics with a single antibiotic to analyze the effect of this mixture on the diversity of the intestinal flora. Our findings provide clinically relevant information for the treatment of bacterial infections and verify the effectiveness of the current methods commonly used to eliminate the intestinal microbiota.

## RESULTS

### Treatment with four antibiotics reduced the richness and diversity of the intestinal microbiota in mice.

**FIG 1:**
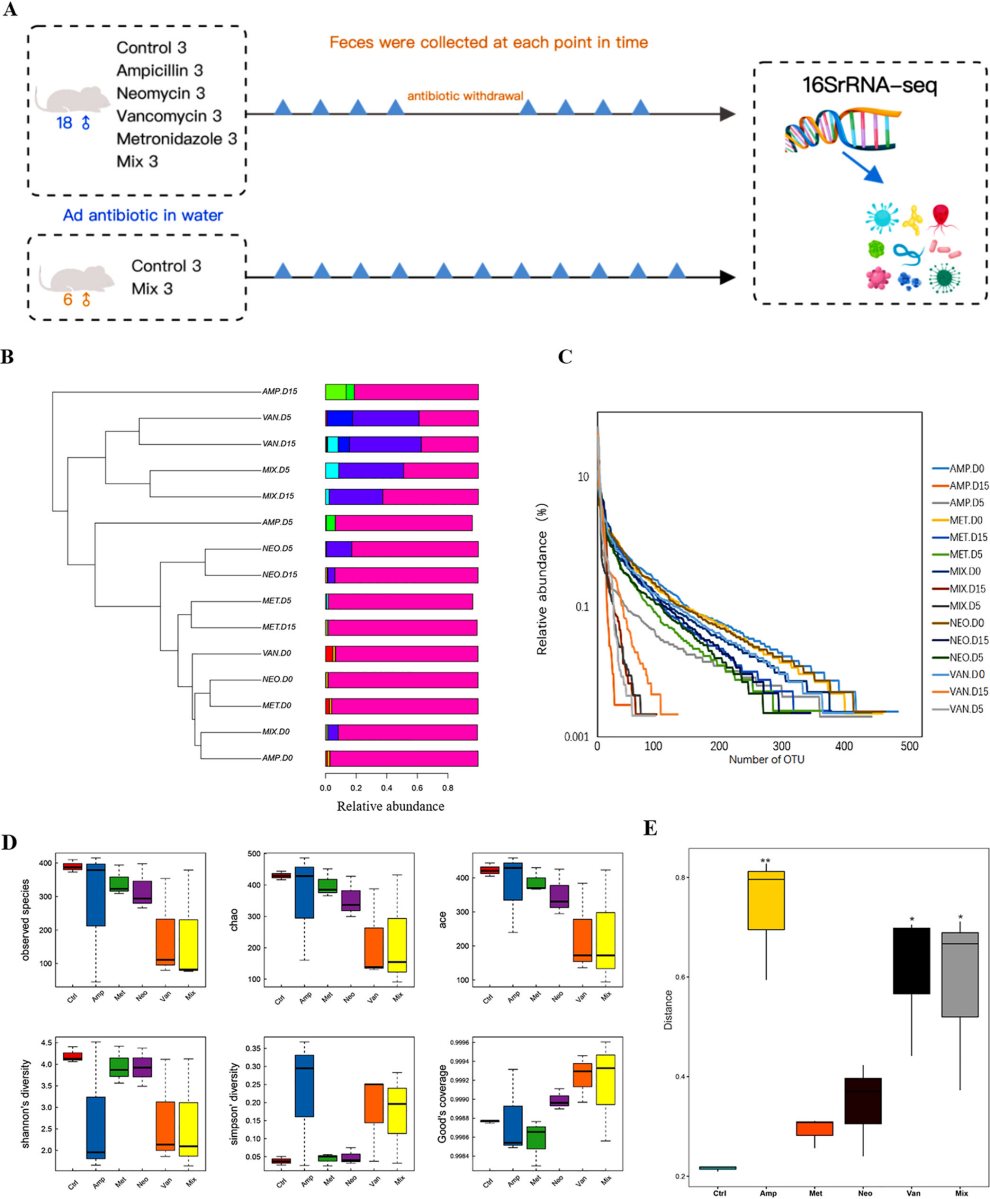
Continuous treatment with four antibiotics alters the diversity and abundance of the intestinal microbiota in mice. (A) Experiment included eight groups of three 18-week-old mice. Groups were treated with 100 mg/kg ampicillin, 50 mg/kg vancomycin, 100 mg/kg metronidazole, or 100 mg/kg neomycin. Fecal samples were collected at eight time points for the short-term experimental groups and at 11 time points for the long-term experimental groups. (B) Met, metronidazole; Amp, ampicillin; Van, vancomycin; Neo, neomycin; Mix, mixture. Microbial UPGMA clustering for fecal samples taken at different time points. Similar samples cluster together on the same branch of the tree. D0 indicates baseline and D indicates different time points. (C) OTU rank curve. Abscissa is sorted by OTU abundance in each sample (high to low), and OTU abundance is on the ordinate axis. Richness of species within the sample is reflected by the horizontal spread of the curve: the wider the curve, the richer the species composition of the sample. (D) Alpha diversity analysis of the microbiota after 14 days of antibiotic treatment. Alpha diversity analysis assesses species diversity within a single sample, and includes the observed species index, Chao index, Ace index, Shannon index, Simpson index, Good’s coverage index, and other indices. (E) Beta diversity. (Kruskal test and Wilcoxon test: *, *P* < 0.05; **, *P* < 0.01).

As shown in Fig. 1A, eight groups of three mice were studied. Antibiotic concentrations were based on the weight of the mice, and antibiotics were continuously administered for 14 days. We collected fecal samples from each group before the start of treatment and on the 5th and 15th days after treatment began, and performed 16S rRNA-seq analysis on DNA isolated from collected fecal material.

First, we carried out an unweighted pair-group method with arithmetic means (UPGMA) cluster analysis to divide the samples into groups based on microbiota composition. Samples with high similarity in terms of microbiota composition were clustered into the same branch of the evolutionary tree. Fig. 1B shows the baseline samples for all of the groups clustered together. After antibiotic treatment was started, the microbiota composition of each group began to change and gradually diverge from the respective baseline samples. Subsequently, we analyzed the impact of the four antibiotic treatments on the diversity of the intestinal microbiota as measured by the number of identified operational taxonomic units (OTUs), as shown in Fig. 1C. The curves gradually fell, indicating that the richness of the microbial diversity was greatly reduced. Alpha diversity analyses assess species diversity within a single sample, and include the observed species index, Chao index, Ace index, Shannon index, Simpson index, and Good’s coverage index. To perform an alpha diversity analysis, we selected the Shannon index. There was no significant difference in the mean Shannon diversity index before and after antibiotic treatment (Fig. 1D; Wilcoxon test, *P* > 0.05), but the microbial richness of the Van, Met, Neo, and S-Mix groups decreased compared to that of the control group. Beta diversity analysis (Fig. 1E) showed that the microbial richness in the Amp (Wilcoxon Test, P<0.01), Van (Wilcoxon Test, P<0.05), and S-Mix (Wilcoxon test, *P* <0.05) groups decreased compared with that of the control group. The antibiotics used to treat these three groups are active against Gram-negative, Gram-positive, and both Gram-negative and Gram-positive bacteria, respectively. Neomycin and metronidazole target aerobic and anaerobic bacteria, respectively, but they had no significant effect on the diversity of intestinal bacteria. In terms of effect on the diversity of the gut microbiota, the combination of four antibiotics was inferior to ampicillin or vancomycin alone. This indicates that the combination of multiple antibiotics did not have the greatest impact on intestinal flora, and that the combination of these four antibiotics is not necessarily the optimal choice for eliminating the intestinal microbiota in experimental mouse models.

### Effects of short-term antibiotic use on the murine intestinal microbiota.

**FIG 2:**
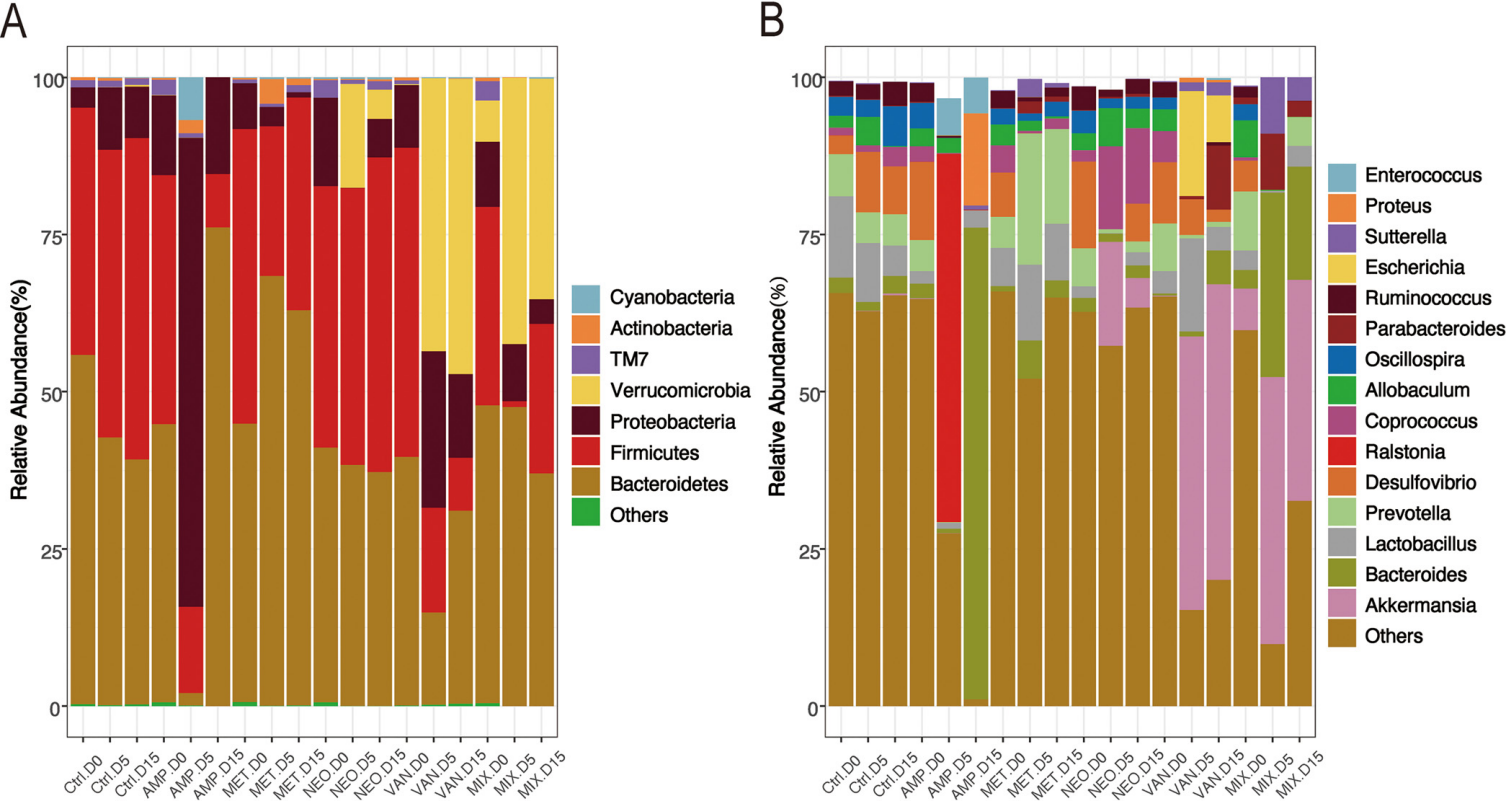
Treatment with the four antibiotics altered the abundance of most taxa. Histograms of species abundance. Sample names are on the abscissa, and relative abundance of the annotated species is shown on the ordinate axis. Species with less than 0.5% abundance in all samples were merged into the ‘Others’ category. (A) Abundance at the phylum level. (B) Abundance at the genus level. (Wilcoxon test: *, *P* < 0.05; **, *P* < 0.01).

Considering the substantial impact of antibiotics on the richness and diversity of the intestinal microbiota, we next decided to study its impact on specific taxa. We compared the treatment group and the control group using the Wilcoxon test at the phylum and genus level (Tables S1 and S2). Fig. 2A and B show that at the phylum level, Proteobacteria increased following ampicillin treatment while TM7 and Firmicutes decreased; at the genus level, *Ruminococcus* and *Coprococcus* of the Firmicutes decreased, while Proteus in Proteobacteria and *Bacteroides* in Bacteroidetes increased. At the phylum level, Verrucomicrobia (*P* < 0.05) and Proteobacteria increased following vancomycin treatment, while Actinobacteria (Wilcoxon test, *P* < 0.05), and TM7 (*P* < 0.05) decreased; at the genus level, *Oscilillospira* (*P* < 0.05), *Allobaculum*, and *Coprococcus* of the Firmicutes decreased, while Proteus in Proteobacteria and *Bacteroides* in Bacteroidetes increased. Following treatment with metronidazole, at the phylum level, Actinobacteria, Bacteroidetes, and Cyanobacteria (Wilcoxon test, *P* < 0.05) increased, while Proteobacteria and Firmicutes decreased; at the genus level, *Coprococcus* of the Firmicutes decreased, while *Sutterella* in Proteobacteria, *Prevotella*, and *Bacteroides* increased. After treatment with neomycin, at the phylum level, Verrucomicrobia increased, while TM7 and Proteobacteria (Wilcoxon test, *P* < 0.05) decreased; it is worth noting that *Allobaculum* and *Coprococcus* (*P* < 0.05) of the Firmicutes increased. After treatment with the mixture, at the phylum level, Actinobacteria (Wilcoxon test, *P* < 0.05) and Verrucomicrobia increased, while Cyanobacteria, TM7 (*P* < 0.05), Firmicutes, and *Proteobacteria* decreased. Additionally, at the genus level, *Ruminococcus*, *Allobaculum*, and *Coprococcus* of the Firmicutes decreased, while *Sutterella* and *Bacteroides* of the Proteobacteria decreased.

In general, some genera showed a decrease in relative abundance, while others showed an increase. *Coprococcus* and *Lactobacillus* are two types of Gram-positive anaerobic bacteria that both contain strains of typical probiotics.

Amp, Met, and Van all reduced the abundance of these two bacteria. On the other hand, *Enterococcus*, a Gram-positive, facultative anaerobic bacterium that contains multiple pathogenic strains, should theoretically have been killed by Amp, Met, and Van; however, treatment with Amp and Van led to an increase in *Enterococcus* abundance, and Met had no major effect on its abundance. These findings suggest that the changes that we observed in the intestinal microbiota reflect not only the mechanisms of action of the antibiotics (Table S3), but also competition between different bacterial communities induced by intestinal microbiota dysbiosis. For example, the abundance of *Coprococcus* and *Lactobacillus*, which both contain a strain of typical probiotics, decreased in the four groups of Amp, Met, Van, and Mix, however, this was accompanied by an increase in the abundance of *Enterococcus*, which contains pathogenic strains. The abundance between the two species did not correlate exclusively with the type of antibiotic used, but showed a similar competitive correlation.

### Recovery of the murine intestinal microbiota after antibiotic withdrawal.

**FIG 3:**
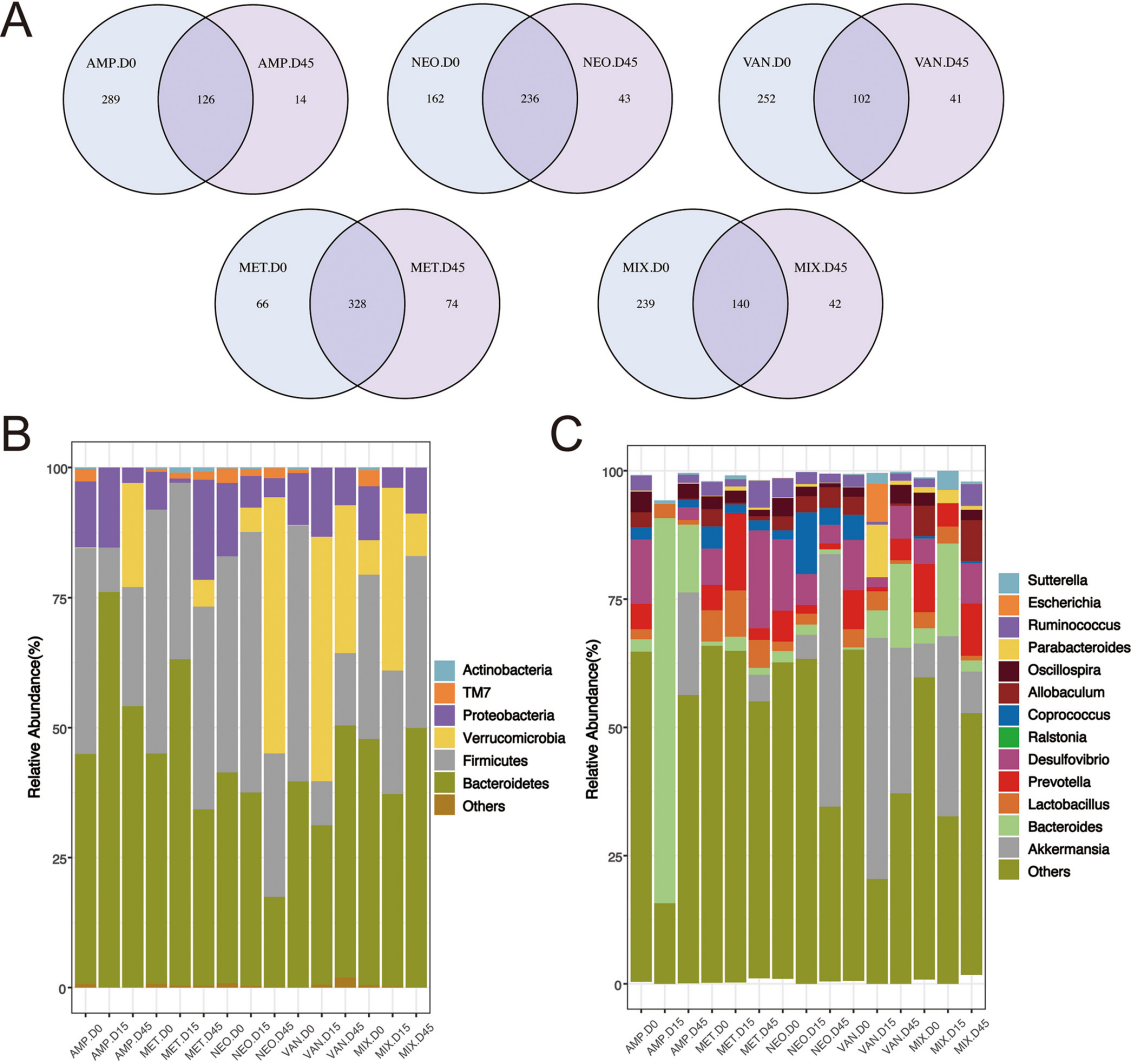
Incomplete and antibiotic-specific recovery of the murine intestinal microbiota after antibiotic withdrawal. (A) Differently colored circles in the Venn diagrams represent different samples or groups. Numbers shown in the overlapping areas indicate the number of OTUs shared between the two samples or groups. Panels B and C show histograms of phylum and genus abundance, respectively. Species whose abundance was less than 0.5% in all samples were merged into the ‘Others’ category.

Next, we investigated the ability of the murine intestinal microbiota to recover 1 month after antibiotics had been withdrawn. We focused on those bacteria whose relative abundance changed significantly during antibiotic treatment. As shown in Fig. 3A, 1 month after the end of antibiotic therapy, the composition of the intestinal microbiota in mice from the Amp, Van, Neo, and S-Mix groups did not return to baseline at the OTU level; only the Met group returned to baseline. At the phylum level, as shown in Fig. 3B, only the abundance of Actinobacteria (D0, D15, D45; 0.28%, 6.23E-03%, and 0.05%, respectively) in the Ampicillin group, and Proteobacteria (10.38%, 3.923%, 8.81%), Bacteroidetes (47.28%, 36.98%, 49.86%), Firmicutes (31.59%, 23.74%, 33.06%), and Verrucomicrobia (6.57%, 35.11%, 8.10%) in the Mixture group, returned to baseline levels, while the previously detected phyla Actinobacteria and Cyanobacteria disappeared. The abundance of only a few genera fully recovered. As shown in Fig. 3C, the abundance of *Ruminococcu*s (2.92%, 3.12E-03%, 1.60%), which had previously decreased, was restored to baseline levels in the Ampicillin group; in the Vancomycin group, the abundance of *Oscillospira* (1.84%, 4.50E-03%, 3.63%) and *Coprococcus* (4.93%, 6.75E-03%, 0%) recovered. *Parabacteroides* and Escherichia, which had not previously reached the detection limit in the Neo group, were detected 1 month after neomycin treatment had stopped. The abundance of *Bacteroides* (2.97%, 18.01%, 2.18%), which had decreased, *Allobaculum* (5.91%, 2.24E-03%, 7.92%), and *Coprococcus* (0.50%, 2.24E-03%, 0.46%), which had increased, returned to baseline levels in the Mixture group. In addition, the abundance of the genera Proteus and *Enterococcus*, which had been detected prior to antibiotic treatment, did not reach the detection limit, and *Ralstonia*, which did not appear prior to treatment, was detected 1 month after withdrawal of the antibiotics.

After antibiotic treatment, the abundance of some bacteria in the intestinal microbiota returned to their baseline levels. However, the abundance of most of the microbiota components was altered, in some cases permanently, and the microbiota composition following antibiotic withdrawal was different from that observed before treatment. Our findings show that while the abundance of some intestinal microbiota components was restored to pretreatment levels, the abundance of most of the intestinal microbiota components did not recover, and instead remained elevated. The microbiota composition of groups treated with a single antibiotic exhibited less recovery than that of the groups which received a mixture of antibiotics. These changes could increase the risk of intestinal infections and promote inflammatory responses. Our findings suggest that patients who have been prescribed antibiotics should also be supplemented with potentially beneficial bacteria to help restore the balance of their intestinal flora as soon as possible. One study found that probiotics are associated with a reduction in antibiotic-associated diarrhea (28, 29).

### Effects of long-term antibiotic use on the murine intestinal microbiota.

**FIG 4:**
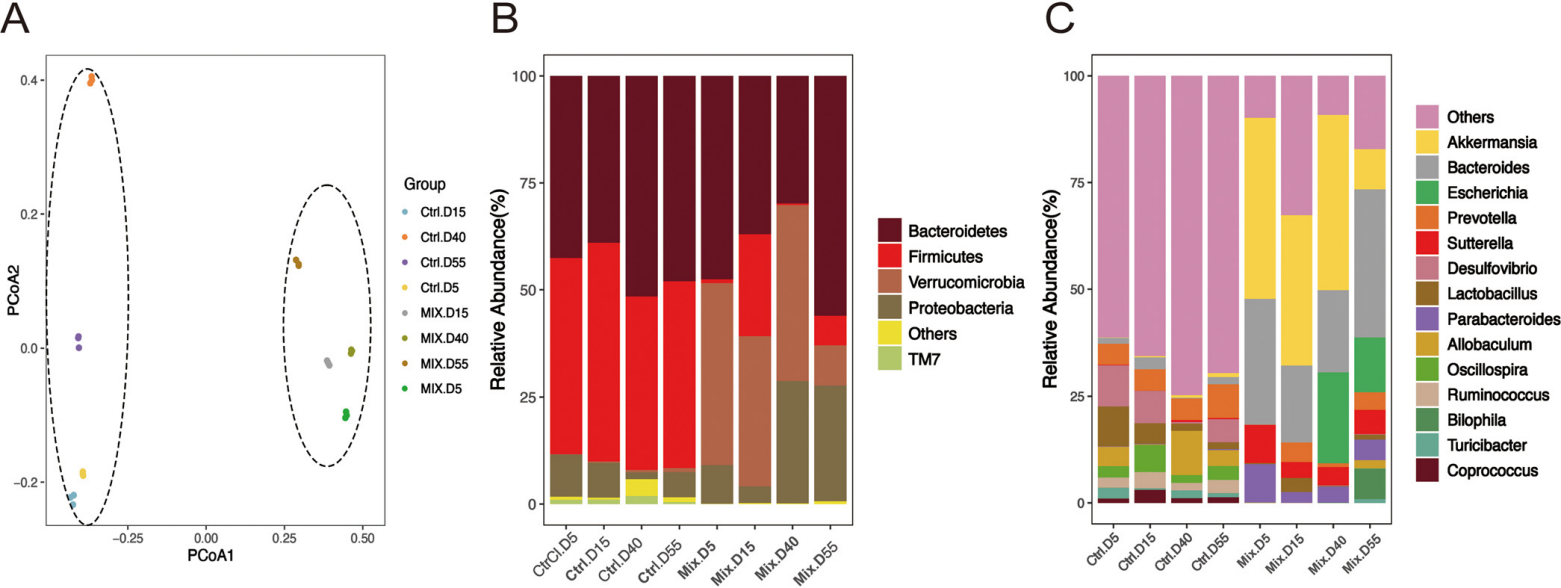
Effects of long-term antibiotic treatment on the composition of the murine intestinal microbiota. (A) PCA analysis. Ctrl, Control group; Mix, Mixture group. The more closely two samples group together on the graph, the more similar their composition. Values for samples taken from different treatment groups or environments can be either dispersed or aggregated, and the degree of dispersion can indicate the degree of similarity between the compositions of samples taken under the same conditions. Panels B and C show histograms of phylum and genus abundance, respectively. Sample names are shown on the abscissa, and relative abundance of the annotated species is shown on the ordinate axis. Species whose abundance was less than 0.5% in all samples were merged into the ‘Others’ category.

In the experiments described above, we analyzed changes in intestinal microbiota diversity and structure induced by short-term antibiotic use. Long-term use of antibiotics exerts different selection pressures on bacteria, inducing drug resistance and leading to increased abundance of opportunistic and pathogenic bacteria. Next, we analyzed the two long-term groups (male mice), which received either normal sterile water (control group) or continuous treatment with a mixture of antibiotics in their drinking water (100 mg/kg ampicillin; 50 mg/kg vancomycin; 100 mg/kg metronidazole; and 100 mg/kg neomycin) for 55 days (L-Mix group). Fecal samples were collected at 11 time points, and four of these were chosen for analysis: days 5, 15, 40, and 55. Principal-component analysis (PCA) (Fig. 4A) showed that the microbiota composition at all four time points from the control group were clustered together, indicating that their composition was very similar. In contrast, the values for the four time points for the L-Mix group were scattered across the graph, indicating that there were relatively large differences in the microbiota composition between these four time points. This indicates that the impact of antibiotics on the intestinal microbiota is continuous, and that the microbiota structure is constantly changing. In addition, as shown in Fig. 4B, at the phylum level, the abundance of Bacteroidetes (D0, D40, D55; 47.54%, 29.78%, and 55.95%, respectively) first decreased and then increased. At the genus level (Fig. 4C), the abundance of *Bacteroides* (4.68%, 19.16%, 34.61%) increased. We speculate that long-term antibiotic use led to the appearance of resistant strains in the intestinal microbiota. When a population of bacteria is exposed to an antibiotic, the frequency of resistant clones will increase over time. We suggest that this effect also applies to the entire microbiome, with various microorganisms differing in their susceptibility to different antibiotics, and that new steady-state levels will be reached as the frequency of some susceptible species decreases or they become extinct (30, 31).

## DISCUSSION

In this study, we used 16S rRNA gene sequencing technology to determine the short-term and long-term effects of ampicillin, vancomycin, metronidazole, and neomycin on the intestinal microbiota of mice by analyzing changes in the relative abundance of the bacteria. We found that treatment with ampicillin, vancomycin, metronidazole, neomycin, and a combination of all four antibiotics decreased the diversity of the microbiota. Treatment with the four antibiotics resulted in a decrease in the abundance of genera containing bacteria with beneficial potential, such as *Coprococcus* and *Lactobacillus*, and an increase in the abundance of genera with pathogenic potential, such as *Enterococcus*. Previous studies have suggested that the decrease in the relative abundance of bacteria with beneficial potential observed after antibiotic use is due to a reduction in the absolute levels of bacteria with beneficial potential because of decreased microbiota diversity (32), and our results are similar to theirs at the genus level. We believe that the changes we observed in the intestinal microbiota reflect not only the antibiotics’ mechanisms of action, but also competition between different bacterial communities induced by intestinal microbiota dysbiosis. However, there are limitations here. Both probiotics and bacteria with pathogenic potential are usually species-dependent. 16S rRNA sequencing does not allow for a confident taxonomic assignment at a species level. Hence, it is imprecise to differentiate between “potentially pathogenic” and “potentially beneficial” microorganisms based on this method. This increase in the abundance of bacteria with pathogenic potential may also enhance the host inflammatory response (33).

We also assessed the recovery of the murine intestinal microbiota 1 month after antibiotic treatment had been stopped. In general, we found that the diversity of the intestinal microbiota recovered within a few weeks after the discontinuation of antibiotic use, although the microbiota composition was different than that prior to antibiotic treatment. Recent studies have found that 4 months after the end of ciprofloxacin or clindamycin treatment, the abundance of only two OTUs changed significantly, and that of the other microbiota components returned to baseline levels (22, 34). Our results are inconsistent with those of a previous study (32), because we found that although the abundance of some bacterial genera recovered to baseline levels, the microbiota composition differed from that observed before antibiotic treatment, and in some cases these changes were permanent. Thus, our data indicate that antibiotics have not only a short-term effect on the host intestinal microbiota, but also a long-term effect.

At the genus level (Fig. 4C), the abundance of *Bacteroides* and Escherichia increased. We speculate that long-term antibiotic used leads to the development of resistant strains, which is consistent with previous studies (35–37). In addition, our results show that pathogens such as *Bacteroides* and Escherichia are more likely to develop resistance.

Our study had other limitations. First, only three mice were included per group, but the changes in the intestinal microbiota caused by the antibiotics were drastic, so these results should be interpreted with caution. Second, the effect of antibiotics on the composition and function of the intestinal microbiota can change depending on the type and dosage of antibiotics used and the route of administration. In this study, we administered antibiotics orally to mice for 2 weeks and did not explore the effects of different routes of administration on the murine intestinal microbiota. Third, we did not explore the mechanisms underlying the changes we observed in the microbiota, which could be useful for designing strategies to restore normal host-microbiota interactions. Further research is needed to identify this mechanism.

In summary, our research shows that oral administration of four antibiotics, ampicillin, vancomycin, metronidazole, and neomycin, changes the composition of the murine intestinal microbiota, disrupts the balance in the intestinal microbiota, and reduces the abundance of genera containing potentially beneficial bacteria, such as *Coprococcus* and *Lactobacillus*, and increases the abundance of genera with pathogenic potential, such as *Enterococcus.* We speculate that these changes increase the risk of host infection. In addition, we found that oral antibiotics can have a long-term negative impact on the microbiota and produce drug-resistant bacteria, as indicated by this observation. This effect is likely due to the host’s having been in a state of imbalance for a prolonged period of time. Use of these antibiotics should be considered carefully before they are prescribed in the clinic, and restoration of the intestinal microbiota through fecal transplantation or probiotic administration should be considered.

## MATERIALS AND METHODS

### Antibiotics.

Ampicillin (A5354), vancomycin (V0045000), metronidazole (M3761), and neomycin (33492) were purchased from Sigma-Aldrich (St. Louis, MO, USA).

### Animals.

Eight-week-old C57BL/6 mice were obtained from Shanghai SLAC Laboratory Animal Co., Ltd. Mice (*n* = 5 per group) were maintained in a specific pathogen-free room in the Experimental Animal Center at Tongji University, in cages with free access to water and food and a 12-h/12-h light/dark cycle. After 10-week of acclimation, the mice were fed in seperate cages. We had a total of 24 cage mice, numbered sequentially, and we randomly generated numbers up to 24 using the Microsoft Excel “RANDBETWEEN” function. The mice were organized into groups based on the generated numbers, and antibiotics were added to the drinking water based on the weight of the mice. There were six short-term experimental groups (male mice): control, Amp (100 mg/kg ampicillin), Van (50 mg/kg vancomycin), Met (100 mg/kg metronidazole), Neo (100 mg/kg neomycin), and S-Mix (100 mg/kg ampicillin, 50 mg/kg vancomycin, 100 mg/kg metronidazole, and 100 mg/kg neomycin). In addition, there were two long-term experimental groups (male mice): control and L-Mix (100 mg/kg ampicillin, 50 mg/kg vancomycin, 100 mg/kg metronidazole, 100 mg/kg neomycin). The Amp, Van, Met, Neo, and S-Mix groups received antibiotics for 14 consecutive days, followed by a 30-day washout period, and finally by antibiotic administration for an additional 14 days. The L-Mix group received antibiotics for 55 days. After an overnight fast at the end of the feeding period, mice were deeply anesthetized using tribromoethanol (Sigma, St. Louis, MO). Blood was taken by removing an eyeball and centrifuged at 1,000 × *g* for 10 min at 4°C to obtain plasma. After the experiment, mice were euthanized by cervical dislocation. There were no adverse events. The ethics committee of Tongji University approved all protocols used in this study.

### Genomic DNA extraction.

Total microbial DNA was extracted using a MagPure Stool DNA KF kit B (Magen, China) according to the manufacturer’s instructions. DNA was quantified with a Qubit Fluorometer using a Qubit dsDNA BR assay kit (Invitrogen, USA), and the quality was checked by running an aliquot on 1% agarose gel.

### Library construction.

The V4 variable region of the bacterial 16s rRNA gene was amplified with degenerate PCR primers: 515F (5′-GTGCCAGCMGCCGCGGTAA-3′) and 806R (5′-GGACTACHVGGGTWTCTAAT-3′). Both primers were tagged with Illumina adapter, pad, and linker sequences. PCR enrichment was performed in a 50-μL reaction mixture containing 30 ng template, both tagged PCR primers, and PCR master mix. The PCR cycling conditions were as follows: 95°C for 3 min; 30 cycles of 95°C for 45 s, 56°C for 45 s, and 72°C for 45 s; and a final extension for 10 min at 72°C. The PCR products were purified using Agencourt AMPure XP beads and eluted in elution buffer. Libraries were evaluated using an Agilent Technologies 2100 Bioanalyzer. The validated libraries were then sequenced on an Illumina HiSeq 2500 platform (BGL, Shenzhen, China) following standard Illumina protocols and generating 2× 250-bp paired-end reads.

### Bioinformatics analysis.

First, low quality was removed from the original sequencing data by the window method with Readfq v8 (https://github.com/cjfields/readfq). Joint pollution reads were removed, n-containing reads were removed, and low-complexity reads were processed. Samples were distinguished based on barcode and primer. After this, we used FLASH software (fast length adjustment of short reads, v1.2.11) (38). Using overlapping relationships, pairs of double-end sequencing reads were assembled into a sequence with high area tags. Effective tags were produced by the UCHIME algorithm and clustered into operational taxonomic units (OTUs) using USEARCH (v7.0.1090) software (39). According to the mothur method and Greengenes database, taxonomic information was annotated with representative sequences from OTUs (40). phytools and R software (v3.4.1) (41) were used to perform an UPGMA clustering analysis based on Bray-Curtis, weighted Unifrac, and unweighted Unifrac distance matrices. The ade4 package of R (v3.4.1) was used to perform an OTU PCA analysis. The RDP classifier Bayesian algorithm was used to classify the OTU representative sequences. The community composition of individual samples was counted at the species level of the phylum, order, family, and genus, and the histogram of species abundance was performed using the ggplot2 package of R (v3.4.1). Alpha diversity statistics were performed using the software motor (v1.31.2). Beta diversity was analyzed by QIIME (v1.80). Finally, the ggplot2 package of R (v3.4.1) was used for box plots of alpha diversity and beta diversity.

### Statistical analysis.

Data are expressed as mean ± standard error of the mean, and *P* values of <0.05 were considered statistically significant. When the number of groups was two, the rank-sum test was used for analysis of variance, and the Wilcoxon rank-sum test was used for two-sample comparisons (in R wilcox.test). When the number of groups was greater than two, the Kruskal-Wallis test for multisample comparisons was used (in R kruskal.test).

### Data availability.

The original 16S rRNA sequence data are available from the NCBI under accession number PRJNA636223.
