# Supplementary material for: Effects of Four Antibiotics on the Diversity of the Intestinal Microbiota
Source: Microbiol Spectr. 2022 Mar 21;10(2):e01904-21. doi: 10.1128/spectrum.01904-21 (PMC9045271; doi:10.1128/spectrum.01904-21)
Supplement: SUPPLEMENTAL FILE 1 — Supplemental material. Download Spectrum01904-21_suppemental_material.doc, DOCX file, 0.08 MB [file spectrum01904-21_suppemental_material.doc]

**Supplementary**

Table S1 Mice intestinal microbiota relative abundance on phylum level

| **Taxon** | **Control** | **Ampicillin** | **Metronidazole** | **Neomycin** | **Vancomycin** | **Mixture** |
| --- | --- | --- | --- | --- | --- | --- |
| ***Cyanobacteria*** | **5.53E-04±5.73E-04** | **3.41E-02±4.81E-02** | **2.59E-03±3.26E-04*** | **3.00E-03±2.34E-04** | **1.68E-03±1.99E-04** | **1.19E-03±1.62E-03** |
| ***Actinobacteria*** | **4.43E-03±1.74E-05** | **1.04E-02±1.46E-02** | **2.47E-02±2.08E-02** | **2.32E-03±1.13E-03** | **3.59E-04±4.78E-04*** | **2.64E-04±3.41E-04*** |
| ***TM7*** | **1.09E-02±9.38E-04** | **3.58E-03±5.06E-03** | **8.13E-03±4.48E-03** | **9.74E-03±5.11E-03** | **1.07E-05±1.51E-05*** | **2.20E-05±3.10E-05*** |
| ***Verrucomicrobia*** | **3.32E-04±4.34E-04** | **1.45E-04±2.05E-04** | **3.81E-05±1.80E-05** | **1.06E-01±8.38E-02** | **4.52E-01±2.48E-02*** | **3.88E-01±5.17E-02** |
| ***Proteobacteria*** | **6.56E-02±4.75E-02** | **4.50E-01±4.18E-01** | **1.96E-02±1.58E-02** | **3.07E-02±4.29E-02*** | **1.90E-01±8.17E-02** | **6.51E-02±3.65E-02** |
| ***Firmicutes*** | **4.25E-01±4.52E-02** | **1.11E-01±3.72E-02** | **2.89E-01±7.10E-02** | **4.70E-01±4.26E-02** | **1.26E-01±5.84E-02** | **1.23E-01±1.62E-01** |
| ***Bacteroidetes*** | **4.90E-01±9.16E-02** | **3.90E-01±5.24E-01** | **6.55E-01±3.92E-02** | **3.78E-01±8.35E-03** | **2.26E-01±1.14E-01** | **4.23E-01±7.46E-02** |
| ***Others*** | **2.38E-03±1.08E-03** | **4.71E-04±6.22E-04** | **9.90E-04±6.46E-04** | **2.36E-04±2.34E-04** | **3.31E-03±1.02E-03** | **1.12E-05±1.58E-05** |

The result between each group and the control group was compared with Wilcoxon test, mean±SD, *, p<0.05; **, p<0.01

Table S2 Mice intestinal microbiota relative abundance on Genus level

| **Taxon** | **Control** | **Ampicillin** | **Metronidazole** | **Neomycin** | **Vancomycin** | **Mixture** |
| --- | --- | --- | --- | --- | --- | --- |
| ***Enterococcus*** | **2.46E-05±0.00E+00** | **5.82E-02±1.05E-03*** | **3.81E-05±5.39E-05** | **2.36E-05±3.34E-05** | **1.66E-03±2.35E-03** | **3.36E-05±4.75E-05** |
| ***Proteus*** | **0.00E+00±0.00E+00** | **7.33E-02±1.04E-01** | **.0000a±0.00E+00** | **1.18E-05±1.67E-05** | **5.52E-03±2.69E-03** | **.0000a±0.00E+00** |
| ***Sutterella*** | **1.58E-03±6.08E-04** | **3.21E-03±4.40E-03** | **1.83E-02±1.55E-02** | **4.36E-04±3.18E-04** | **1.73E-02±5.06E-03** | **6.35E-02±3.68E-02** |
| ***Escherichia*** | **2.46E-05±0.00E+00** | **3.62E-04±5.12E-04** | **8.89E-05±1.80E-05** | **.0000a±0.00E+00** | **1.21E-01±6.75E-02** | **1.12E-05±1.58E-05** |
| ***Ruminococcus*** | **2.31E-02±6.59E-04** | **1.59E-03±2.20E-03** | **1.06E-02±5.45E-03** | **1.73E-02±8.44E-03** | **2.72E-03±3.85E-03** | **1.12E-05±1.58E-05*** |
| ***Parabacteroides*** | **1.52E-03±5.21E-04** | **3.27E-04±1.99E-04** | **1.34E-02±7.81E-03** | **3.75E-03±1.60E-03** | **5.35E-02±6.85E-02** | **5.77E-02±4.53E-02** |
| ***Oscillospira*** | **2.85E-02±2.36E-03** | **2.43E-04±3.00E-04*** | **1.79E-02±8.26E-03** | **1.71E-02±2.36E-03** | **3.32E-05±1.67E-05*** | **2.22E-05±2.83E-07*** |
| ***Allobaculum*** | **3.22E-02±1.87E-02** | **1.19E-02±1.69E-02** | **9.52E-03±8.97E-03** | **4.60E-02±2.09E-02** | **1.07E-05±1.51E-05** | **5.93E-04±8.07E-04** |
| ***Coprococcus*** | **1.12E-02±1.53E-03** | **6.52E-04±9.21E-04*** | **1.04E-02±9.19E-03** | **1.26E-01±8.47E-03** | **4.45E-05±3.26E-05*** | **2.22E-05±2.83E-07*** |
| ***Ralstonia*** | **1.23E-05±1.74E-05** | **2.93E-01±4.13E-01** | **0.0000±0.00E+00** | **1.18E-05±1.67E-05** | **0.00E+00±0.00E+00** | **4.48E-05±6.34E-05** |
| ***Desulfovibrio*** | **6.32E-02±4.71E-02** | **2.07E-04±2.93E-04** | **1.40E-04±8.99E-05** | **3.00E-02±4.25E-02** | **3.81E-02±2.65E-02** | **2.20E-05±3.10E-05** |
| ***Prevotella*** | **5.79E-02±1.29E-02** | **3.83E-04±5.41E-04** | **1.80E-01±4.13E-02** | **1.16E-02±7.63E-03** | **6.83E-03±1.92E-03** | **2.30E-02±3.21E-02** |
| ***Lactobacillus*** | **1.12E-01±2.54E-02** | **1.83E-02±1.27E-02** | **1.06E-01±2.14E-02** | **1.08E-02±1.44E-02** | **9.26E-02±7.83E-02** | **1.79E-02±2.15E-02** |
| ***Bacteroides*** | **1.92E-02±7.33E-03** | **3.79E-01±5.25E-01** | **4.42E-02±2.37E-02** | **1.66E-02±4.98E-03** | **3.09E-02±3.24E-02** | **2.37E-01±8.02E-02** |
| ***Akkermansia*** | **3.32E-04±4.34E-04** | **1.45E-04±2.05E-04** | **3.81E-05±1.80E-05** | **1.06E-01±8.38E-02** | **4.52E-01±2.48E-02** | **3.88E-01±5.17E-02** |
| ***Others*** | **6.42E-01±2.04E-02** | **1.43E-01±1.87E-01** | **5.85E-01±9.12E-02** | **6.03E-01±4.30E-02** | **1.77E-01±3.39E-02*** | **2.12E-01±1.61E-01** |

The result between each group and the control group was compared with Wilcoxon test, mean±SD, *, p<0.05; **, p<0.01

Table S3 Types of Antibiotics

| **Antibiotic** | **Dose** | **Spectrum** |
| --- | --- | --- |
| Ampicillin | 100 mg/kg(1, 2) | Gram-positive & Gram-negative bacteria(3) |
| Neomycin | 100 mg/kg | Gram-positive aerobic bacteria & Gram-negative aerobic bacteria(4, 5) |
| Metronidazole | 100 mg/kg | Gram-positive anaerobic bacteria  & Gram-negative anaerobic bacteria(6) |
| Vancomycin | 50 mg/kg | Gram-positive bacteria(7) |

**REFERENCES**

1. Le Roy T, Debédat J, Marquet F, Da-Cunha C, Ichou F, Guerre-Millo M, Kapel N, Aron-Wisnewsky J, Clément K. 2018. Comparative Evaluation of Microbiota Engraftment Following Fecal Microbiota Transfer in Mice Models: Age, Kinetic and Microbial Status Matter. Front Microbiol 9:3289.

2. Miao Z, Lai Y, Zhao Y, Chen L, Zhou J, Li C, Lan H. 2020. Scutellarein Aggravated Carbon Tetrachloride-Induced Chronic Liver Injury in Gut Microbiota-Dysbiosis Mice. Evid Based Complement Alternat Med 2020:8811021.

3. Zhang C, Peng Y, Mu C, Zhu W. 2018. Ileum terminal antibiotic infusion affects jejunal and colonic specific microbial population and immune status in growing pigs. J Anim Sci Biotechnol 9:51.

4. Ghosh SS, Bie J, Wang J, Ghosh S. 2014. Oral supplementation with non-absorbable antibiotics or curcumin attenuates western diet-induced atherosclerosis and glucose intolerance in LDLR-/- mice--role of intestinal permeability and macrophage activation. PLoS One 9:e108577.

5. Hagan T, Cortese M, Rouphael N, Boudreau C, Linde C, Maddur MS, Das J, Wang H, Guthmiller J, Zheng NY, Huang M, Uphadhyay AA, Gardinassi L, Petitdemange C, McCullough MP, Johnson SJ, Gill K, Cervasi B, Zou J, Bretin A, Hahn M, Gewirtz AT, Bosinger SE, Wilson PC, Li S, Alter G, Khurana S, Golding H, Pulendran B. 2019. Antibiotics-Driven Gut Microbiome Perturbation Alters Immunity to Vaccines in Humans. Cell 178:1313-1328.e13.

6. Murgia D, Angellotti G, D'Agostino F, De Caro V. 2019. Bioadhesive Matrix Tablets Loaded with Lipophilic Nanoparticles as Vehicles for Drugs for Periodontitis Treatment: Development and Characterization. Polymers (Basel) 11.

7. Liévin-Le Moal V, Servin AL. 2014. Anti-infective activities of lactobacillus strains in the human intestinal microbiota: from probiotics to gastrointestinal anti-infectious biotherapeutic agents. Clin Microbiol Rev 27:167-99.
